# Daytime plasma cortisol and cortisol response to dexamethasone suppression are associated with a prothrombotic state in hypertension

**DOI:** 10.3389/fendo.2024.1397062

**Published:** 2024-05-21

**Authors:** Gabriele Brosolo, Andrea Da Porto, Luca Bulfone, Antonio Vacca, Nicole Bertin, Cinzia Vivarelli, Cristiana Catena, Leonardo A. Sechi

**Affiliations:** ^1^ Internal Medicine and European Hypertension Excellence Center, Department of Medicine, University of Udine, Udine, Italy; ^2^ Diabetes and Metabolism Unit, Department of Medicine, University of Udine, Udine, Italy; ^3^ Thrombosis and Hemostasis Unit, Department of Medicine, University of Udine, Udine, Italy

**Keywords:** coagulation, cortisol, d-dimer, dexamethasone, fibrinogen, fibrinolysis, prothrombin fragment 1 + 2, von Willebrand factor

## Abstract

**Background and aims:**

A prothrombotic state was demonstrated in patients with Cushing’s syndrome and is involved in the development and progression of cardiovascular and renal damage in hypertensive patients. This study was designed to examine the relationships between cortisol secretion and the hemostatic and fibrinolytic systems in hypertension.

**Methods:**

In 149 middle-aged, nondiabetic, essential hypertensive patients free of cardiovascular and renal complications, we measured hemostatic markers that express the spontaneous activation of the coagulation and fibrinolytic systems and assessed daily cortisol levels (8 AM, 3 PM, 12 AM; area under the curve, AUC-cortisol) together with the cortisol response to dexamethasone overnight suppression (DST-cortisol).

**Results:**

Plasma levels of D-dimer (D-dim), prothrombin fragment 1 + 2 (F1 + 2), and von Willebrand factor (vWF) were progressively and significantly higher across tertiles of AUC-cortisol and DST-cortisol, whereas no differences were observed in fibrinogen, tissue plasminogen activator, plasminogen activator inhibitor-1, antithrombin III, protein C, and protein S. D-dim, F1 + 2, and vWF were significantly and directly correlated with age and both AUC-cortisol and DST-cortisol. Multivariate regression analysis showed that both AUC-cortisol and DST-cortisol were related to plasma D-dim, F1 + 2, and vWF independently of age, body mass index, blood pressure, and renal function.

**Conclusion:**

Greater daily cortisol profile and cortisol response to overnight suppression are independently associated with a prothrombotic state in hypertensive patients and might contribute to the development of organ damage and higher risk of cardiovascular complications.

## Introduction

Epidemiological evidence indicates that the coagulation system plays an important role in the pathophysiology of atherosclerosis (1), and increased incidence of cardiovascular events has been associated with elevated circulating levels of markers of hemostatic activation (2–4). A prothrombotic state is characterized by intrinsic subclinical activation of the hemostatic system and is a well-recognized risk factor for cardiovascular events in the general population (5, 6) and in patients with hypertension (7, 8) and early renal failure (9). In hypertension, a prothrombotic state is associated with subclinical changes of the arterial tree (10–12), impaired left ventricular diastolic properties (13), and intrarenal hemodynamic changes (14) that are associated with decreased glomerular filtration rate (15). Furthermore, a link of the prothrombotic state with an activated renin-angiotensin-aldosterone system has been demonstrated in hypertension (16, 17) suggesting interaction of the hemostatic system with blood pressure regulatory mechanisms.

A prothrombotic state can be detected in many disease states including those characterized by exogenous or endogenous hypercortisolism (18). In patients with Cushing’s syndrome, elevated circulating levels of a multitude of hemostatic markers were demonstrated, together with evidence of an impaired fibrinolytic system (19–21). Interestingly, initial studies reported activation of the hemostatic system also in patients with subclinical Cushing’s syndrome (22, 23) such as those with incidentally detected adrenal masses in the absence of overt clinical features of hypercortisolism (24). Along these lines, studies conducted in other groups of patients have reported observations that might suggest the existence of a relationship between circulating glucocorticoids and different components of the hemostatic and fibrinolytic systems (25–27).

Although patients with essential hypertension have cortisol secretory rates and response to overnight dexamethasone suppression (DST) within the physiologic range, minor differences in circulating cortisol levels might have some impact on the coagulation system. To date, no information is available on the possible relationship of circulating cortisol with the hemostatic-fibrinolytic balance in patients with hypertension. This information would be relevant for better understanding of mechanisms that contribute to the development and progression of hypertensive organ damage.

The aim of this study was to test the hypothesis that cortisol secretory rates and response to DST are associated with changes in the activity of the coagulation system. We examined the relationship of cortisol daily production and response to DST with a broad panel of hemostatic markers in nondiabetic patients with hypertension who were free of major cardiovascular and renal complications.

## Materials and methods

### Patients

Patients with grade 1/grade 2 essential hypertension who consecutively presented at the Hypertension Clinic of our department from January 2021 to December 2021 were included in a cross-sectional study. All patients were white Caucasian, lived in the North-East of Italy, and were representative of the hypertensive population of this regional territory (28). Blood pressure was measured in patients who remained supine for at least 15 minutes using an automatic tool equipped with appropriately sized cuffs (Omron M6, OMRON Healthcare Co., Kyoto, Japan), obtaining 3 separate readings. Diagnosis of hypertension was done after measurements obtained in at least 3 separate visits, according to current guidelines (29). We excluded patients with: age <18 or >80 years; body mass index (BMI) >40 kg/m^2^; pregnancy or use of estrogens; any type of treatment with corticosteroids; history of alcohol abuse; grade 3 hypertension and secondary hypertension; diabetes; major depressive disorders; 24-hour creatinine clearance <60 ml/min/1,73 m^2^; use of any type of drugs that could interfere with the hemostatic system; history of recent illness and acute or chronic inflammatory conditions; history of cerebrovascular, ischemic heart, or peripheral artery disease. Causes of secondary hypertension were ruled out according to current guidelines (29) as previously reported (30). Cushing’s syndrome was excluded by measurement of daily plasma cortisol (8 AM, 3 PM, 12 AM), 24-hour free urinary cortisol, and plasma cortisol after an overnight DST with 1 mg dexamethasone, according to guidelines (31). When plasma cortisol after DST was >50 nmol/L (15 patients) a 2 mg/48-hour dexamethasone test was done to confirm suppression of cortisol below that level. All patients underwent either MRI or CT of adrenals to exclude presence of incidentalomas. Diabetes was excluded by measurement of fasting blood glucose and glycated hemoglobin, and by a standard oral glucose tolerance test (32). Smokers were defined if they smoked for more than 5 years and did not quit more than 1 year before examination. A standardized questionnaire was used to assess daily alcohol intake (33). The study was conducted following the statements of the Declaration of Helsinki and was approved by the Institutional Review Board of the Department of Medicine. All patients gave their informed consent.

### Laboratory tests

Blood was collected by venipuncture without venous stasis in the early morning after an overnight fast. Plasma was separated and stored at -80°C until processing. Glucose, total and high-density lipoprotein cholesterol, triglycerides were measured with chemical methods in automated devices, as previously reported (34). Low-density lipoprotein level was calculated by the Friedewald formula. Glomerular filtration rate was assessed by duplicate measurements of 24-hour creatinine clearance. Coagulation parameters were measured in plasma as previously described (35). In brief, fibrinogen was assayed in an automatic coagulometer by a functional test, D-dimer was assayed immunoenzymatically, prothrombin fragment 1 + 2 (F1 + 2) and tissue-plasminogen activator (tPA) by an enzyme-linked immunosorbent assay, plasminogen activator inhibitor-1 (PAI-1) by immunoassay, antithrombin III (AT-III), protein C, protein S, and von Willebrand factor (vWF) by functional chromogenic assays. Plasma cortisol was measured by immunoassay (Electro Chemiluminescence ECLIA, Elecsys cortisol II, Roche Diagnostics, Basel, Switzerland) with an intraassay and interassay coefficient of variation of 1.7% and 2.3%, respectively, and the lowest detection limit of 1.5 nmol/L. The area under the curve of daily cortisol (8 AM, 3 PM, 12 AM; AUC-cortisol) was calculated by the trapezoidal rule (36). Duplicate 24-hour urinary collections were obtained for measurement of free cortisol excretion with a direct chemiluminescence technique (ADVIA Centaur Cortisol Immunoassay System, Siemens Healthcare, Milan, Italy; detection range, 14–2069 nmol/l) and the average value was considered.

### Statistical analysis

The Kolmogorov-Smirnov test was used to determine normality of distribution of the variables included in the study. Normally distributed variables are expressed as mean ± standard deviation and skewed variables as median [interquartile range]. Categorical data are expressed as absolute number and percentage. For statistical reasons patients were grouped in tertiles of either AUC-cortisol or DST-cortisol and two-way ANOVA and the Kruskal-Wallis test were used for comparisons among groups with normal or skewed variable distribution, respectively. The Pearson’s chi-square test was used to compare frequency distributions. The relationships between different variables were examined by linear regression analysis, and correlation was expressed by the correlation coefficient *r*. In this analysis, variables with skewed distribution were log transformed. Multivariate regression analysis was performed to determine which variables were independently associated with hemostatic markers. A P value of less than 5% was considered to indicate statistical significance. All data analyses were performed using Stata 12.1 (StataCorp LP, College Station, TX, USA).

## Results

One-hundred-forty-nine patients (age, 48±13 years; 77 males, 72 females) with essential hypertension were recruited for data analysis. Forty (27%) patients had BMI >30 of whom 21 (14%) had grade 1 (BMI 30–35) and 19 (13%) grade 2 obesity. Sixty-two (42%) patients had never been treated with antihypertensive agents and the remaining 87 (58%) who were taking an average of 1.3 antihypertensive agents (angiotensin-converting enzyme inhibitors or angiotensin receptor antagonists, 39%; calcium-channel blockers, 38%; diuretics, 19%; beta-blockers, 19%; alpha-blockers, 7%) had their drugs withdrawn for at least two weeks before the study.

For statistical reasons, patients were grouped according to tertiles of AUC-cortisol and DST-cortisol. The clinical characteristics of the study patients are summarized in **Table 1** together with general biochemistries. Across AUC-cortisol and DST-cortisol tertiles, no significant differences were observed in demographic and anthropometric characteristics, systolic and diastolic blood pressure, plasma glucose and glycated hemoglobin, plasma lipids, electrolyte levels, renal function, and C-reactive protein. No differences were also observed in frequency of previous use of antihypertensive drugs. In particular, no significant differences were observed from patients previously treated with diuretics and the remaining patients. **Table 2** summarizes hormonal measurements of the study patients showing that plasma cortisol levels measured at 8 AM, 3 PM, and 12 AM increased significantly across AUC-cortisol and DST-cortisol tertiles, whereas plasma ACTH, active renin and aldosterone, and daily urinary excretion of epinephrine, norepinephrine, and dopamine did not differ among groups. **Table 3** shows the hemostatic variables showing that plasma D-dimer, F1 + 2, and vWF levels were significantly and progressively higher across both AUC-cortisol and DST-cortisol tertiles. No significant differences among groups were observed in fibrinogen, t-PA, PAI-1, AT-III, and protein C and protein S.

**Table 1:** Clinical Characteristics and biochemical variables of hypertensive patients who were grouped according to either tertiles of the area under the curve (AUC) of daily plasma cortisol or plasma cortisol after dexamethasone suppression (DST).

Variables	All patients (n=149)	AUC Tertile I (n=49)	AUC Tertile II (n=50)	AUC Tertile III (n=50)	DST Tertile I (n=49)	DST Tertile II (n=50)	DST Tertile III (n=50)
*Clinical characteristics*							
Age, years	48 ± 13	48 ± 13	48 ± 13	49 ± 13	46 ± 10	48 ± 13	50 ± 15
Males, n (%)	77 (52)	22 (45)	28 (56)	27 (54)	27 (55)	25 (50)	25 (50)
Body mass index, kg/m^2^	28.8 ± 5.8	28.2 ± 4.6	29.2 ± 6.4	29.1 ± 5.9	29.8 ± 6.4	28.5 ± 5.5	28.2 ± 4.9
Systolic BP, mm Hg	154 ± 17	154 ± 17	155 ± 18	152 ± 17	151 ± 17	154 ± 16	156 ± 19
Diastolic BP, mm Hg	95 ± 11	96 ± 9	94 ± 9	95 ± 14	96 ± 8	95 ± 13	93 ± 12
Previous drug use, n (%)	87 (58)	26 (53)	31 (62)	30 (60)	31 (63)	26 (52)	30 (60)
Alcohol intake, g/day	6 ± 12	5 ± 8	9 ± 14	8 ± 18	6 ± 16	5 ± 11	6 ± 10
Smokers, n (%)	29 (19)	8 (16)	11 (22)	10 (20)	10 (20)	11 (22)	8 (16)
*Biochemical variables*							
Glucose, mg/dL	89 ± 11	87 ± 9	90 ± 11	91 ± 12	88 ± 9	89 ± 10	91 ± 14
Glycated hemoglobin, %	5.6 ± 0.4	5.6 ± 0.5	5.5 ± 0.3	5.7 ± 0.5	5.5 ± 0.4	5.6 ± 0.4	5.7 ± 0.5
Triglycerides, mg/dL	116 ± 66	101 ± 57	124 ± 61	123 ± 78	124 ± 72	107 ± 51	118 ± 73
Total cholesterol, mg/dL	195 ± 40	193 ± 40	190 ± 37	200 ± 42	194 ± 41	197 ± 35	192 ± 44
HDL-cholesterol, mg/dL	55 ± 16	55 ± 15	55 ± 18	56 ± 15	53 ± 14	56 ± 16	56 ± 17
LDL-cholesterol, mg/dL	115 ± 35	117 ± 34	111 ± 34	117 ± 37	116 ± 30	117 ± 26	113 ± 39
GFR, ml/min/1.73 m^2^	103 ± 26	106 ± 25	106 ± 26	98 ± 25	105 ± 31	104 ± 23	100 ± 23
Sodium, mmol/L	141 ± 2	142 ± 2	142 ± 3	141 ± 2	141 ± 2	141 ± 2	141 ± 2
Potassium, mmol/L	4.06 ± 0.42	4.10 ± 0.43	4.06 ± 0.31	4.01 ± 0.49	4.14 ± 0.40	4.04 ± 0.32	3.99 ± 0.50
C-reactive protein, mg/L	1.6 [0.7–3.7]	1.5 [0.8–3.1]	1.3 [0.6–3.1]	1.9 [0.9–4.1]	1.7 [0.8–3.9]	1.4 [0.7–2.5]	1.8 [0.8–4.0]

Values are expressed as mean ± SD. Median and interquartile range in square brackets are shown for variables with skewed distribution. Two-way ANOVA and the Kruskal-Wallis test were used for comparisons among groups with normal or skewed variable distribution, respectively. The Pearson’s chi-square test was used to compare frequency distributions. BP, blood pressure; HDL, high-density lipoproteins; LDL, low-density lipoproteins; GFR, glomerular filtration rate as assessed by 24-hour creatinine clearance.

**Table 2:** Hormonal variables of hypertensive patients who were grouped according to either tertiles of the area under the curve (AUC) of daily plasma cortisol or plasma cortisol after dexamethasone suppression (DST).

Variables	All patients (n=149)	AUC Tertile I (n=49)	AUC Tertile II (n=50)	AUC Tertile III (n=50)	P	DST Tertile I (n=49)	DST Tertile II (n=50)	DST Tertile III (n=50)	P
Cortisol 8 AM, nmol/L	433 ± 148	366 ± 123	451 ± 131	487 ± 164	<0.001	383 ± 139	430 ± 131	486 ± 158	0.002
Cortisol 3 PM, nmol/L	230 ± 108	164 ± 83	206 ± 54	316 ± 112	<0.001	208 ± 99	218 ± 99	259 ± 119	0.043
Cortisol 12 AM, nmol/L	114 ± 85	78 ± 45	124 ± 105	145 ± 87	<0.001	81 ± 54	122 ± 112	136 ± 73	0.004
AUC-cortisol, nmol/L/d	241 ± 77	163 ± 30	234 ± 23	324 ± 58	<0.001	236 ± 85	234 ± 68	253 ± 78	0.023
U cortisol, nmol/d	719 ± 353	714 ± 373	697 ± 313	745 ± 378	0.792	683 ± 336	724 ± 354	749 ± 375	0.649
DST-cortisol, nmol/L	24 [17–33]	21 [17–32]	25 [18–31]	27 [18–44]	0.026	15 [14–17]	24 [21–28]	43 [33–52]	<0.001
ACTH, pg/mL	21 ± 17	19 ± 17	21 ± 17	23 ± 17	0.506	21 ± 10	22 ± 20	21 ± 19	0.944
Active renin, mUI, mL	8.0 [3.8–17.6]	7.8 [3.2–20.7]	7.8 [3.6–11.1]	9.4 [4.6–17.8]	0.659	9.1 [3.3–17.8]	9.9 [4.2–19.0]	7.7 [3.2–12.1]	0.563
Aldosterone, pg/mL	131 ± 80	131 ± 66	121 ± 74	141 ± 95	0.454	141 ± 91	127 ± 64	125 ± 84	0.561
U epinephrine, nmol/d	22[13–35]	22[11–35]	24[14–34]	21[14–37]	0.904	22[13–33]	24[13–43]	20[13–35]	0.309
U norepinephrine, nmol/d	218 [147–321]	201 [140–266]	238 [139–349]	218 [159–305]	0.278	234 [154–386]	211 [143–326]	211 [128–239]	0.650
U dopamine, nmol/d	1736 [1224–2221]	1770 [1226–2134]	1844 [1398–2376]	1590 [1097–2208]	0.138	1840 [1356–2437]	1806 [1207–2413]	1587 [988–2062]	0.518

Values are expressed as mean ± SD. Median and interquartile range in square brackets are shown for variables with skewed distribution. Two-way ANOVA and the Kruskal-Wallis test were used for comparisons among groups with normal or skewed variable distribution, respectively. U, 24-hour urinary excretion.

**Table 3:** Hemostatic variables of hypertensive patients who were grouped according to either tertiles of the area under the curve (AUC) of daily plasma cortisol or plasma cortisol after dexamethasone suppression (DST).

Variables	All patients (n=149)	AUC Tertile I (n=49)	AUC Tertile II (n=50)	AUC Tertile III (n=50)	P	DST Tertile I (n=49)	DST Tertile II (n=50)	DST Tertile III (n=50)	P
Fibrinogen, mg/dL	344 ± 97	335 ± 92	344 ± 78	353 ± 118	0.656	346 ± 94	338 ± 87	348 ± 111	0.866
D-dimer, ng/mL	206 [172–306]	185 [150–276]	206 [171–275]	253 [203–356]	0.018	191 [148–257]	198 [161–273]	266 [210–349]	0.001
F1 + 2, pmol/L	208 ± 78	182 ± 63	215 ± 82	226 ± 84	0.014	167 ± 59	225 ± 72	230 ± 87	<0.001
vWF antigen, %	123 ± 45	112 ± 38	124 ± 40	135 ± 51	0.034	99 ± 35	129 ± 35	141 ± 53	<0.001
t-PA, ng/mL	5.8 [4.2–7.4]	5.9 [3.9–7.3]	5.8 [3.9–7.7]	6.1 [4.5–7.4]	0.696	5.9 [4.8–7.3]	5.0 [4.0–7.0]	6.4 [3.7–8.4]	0.451
PAI-1, ng/mL	11.4 [5.7–18.1]	10.2 [4.3–18.9]	11.2 [7.0–17.7]	14.0 [6.6–17.9]	0.529	13.8 [8.0–24.6]	11.4 [5.8–16.7]	9.7 [5.4–17.9]	0.100
Antithrombin III, %	102 ± 16	100 ± 12	106 ± 22	100 ± 11	0.095	101 ± 13	101 ± 11	104 ± 22	0.562
Protein C, %	111 ± 22	112 ± 20	109 ± 20	113 ± 25	0.635	115 ± 23	110 ± 18	108 ± 25	0.275
Protein S, %	95 ± 23	92 ± 19	93 ± 18	98 ± 30	0.381	99 ± 28	94 ± 21	91 ± 19	0.220

Values are expressed as mean ± SD. Median and interquartile range in square brackets are shown for variables with skewed distribution. Two-way ANOVA and the Kruskal-Wallis test were used for comparisons among groups with normal or skewed variable distribution, respectively. F1 + 2, prothrombin factor 1 + 2; vWF, von Willebrand factor; t-PA, tissue plasminogen activator; PAI-1, plasminogen activator inhibitor-1.

**Figure 1:**
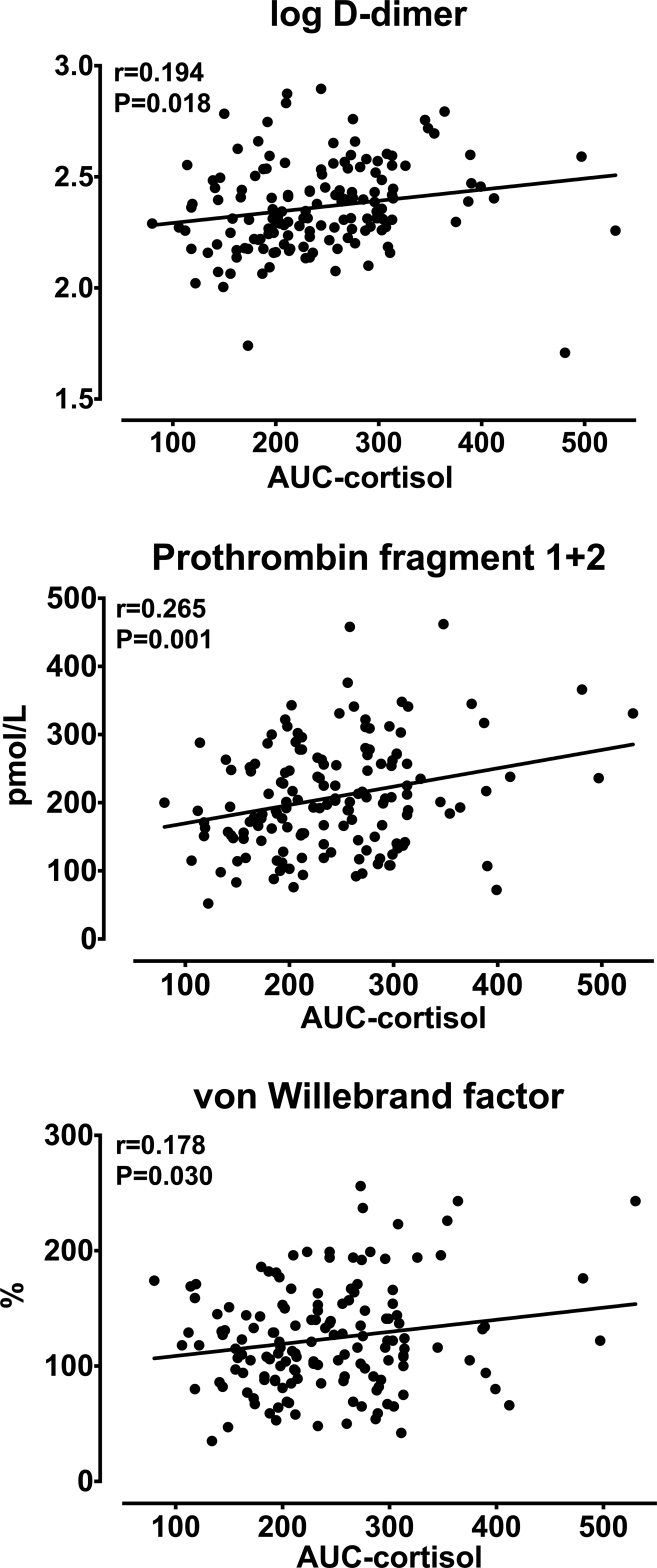
Relationships between the area under the curve (AUC) of daily (8 AM, 3 PM, 12 AM) plasma cortisol and log-transformed D-dimer, prothrombin fragment 1 + 2, and von Willebrand factor.

**Figure 2:**
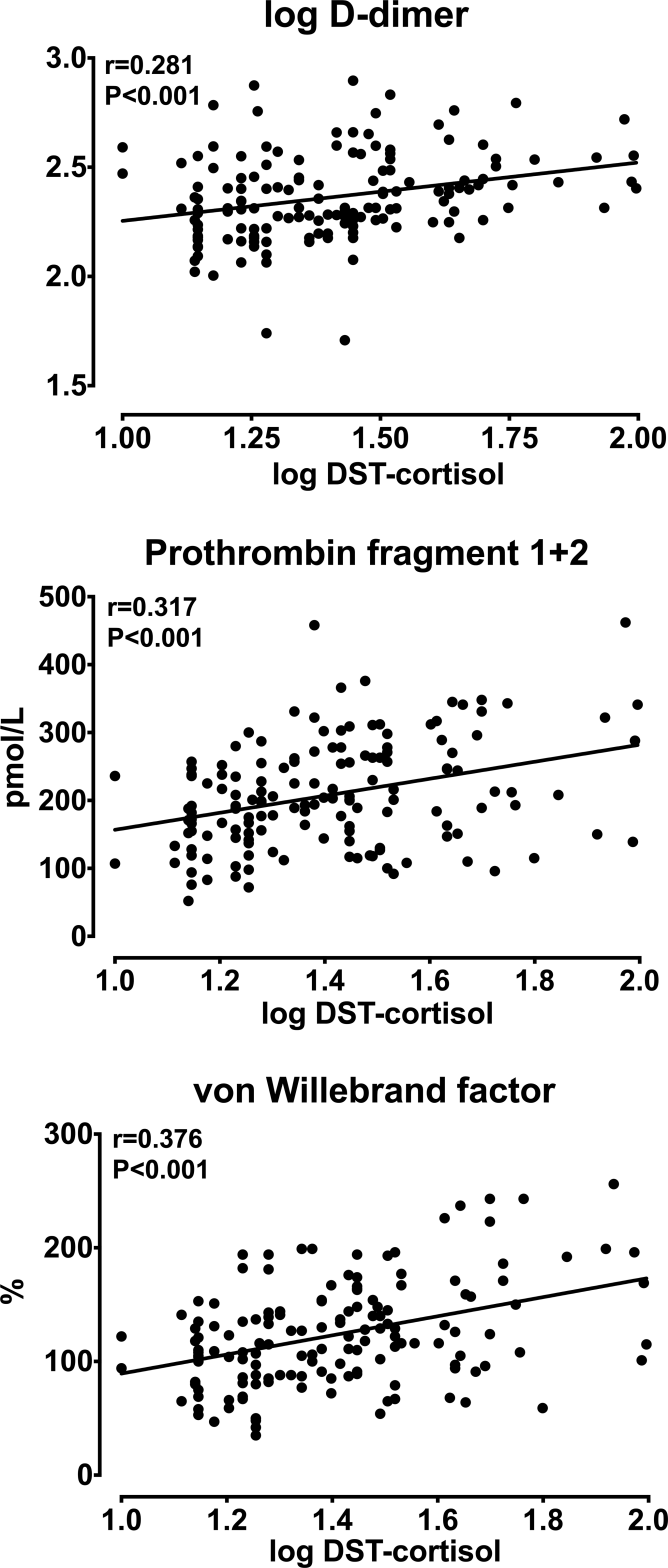
Relationships between log-transformed plasma cortisol measured after a 1 mg overnight dexamethasone suppression test (DST-cortisol) and log-transformed D-dimer, prothrombin fragment 1 + 2, and von Willebrand factor.

Analysis of univariate correlations (**Table 4**) showed that D-dimer, F1 + 2, and vWF were significantly and directly correlated with patients’ age, AUC-cortisol (**Figure 1**) and DST-cortisol (**Figure 2**). No further significant relationship of plasma cortisol levels was observed with the other hemostatic markers. F1 + 2 was also inversely correlated with 24-hour creatinine clearance while fibrinogen, tPA and PAI-1 were directly correlated with BMI. Only fibrinogen was directly correlated with systolic blood pressure. None of the cortisol and hemostatic variables was correlated with C-reactive protein.

**Table 4:** Univariate correlations of clinical and hormonal variables with the hemostatic markers.

Variables	Fibrinogen		D-dimer		F1 + 2		vWf		tPA		PAI-1	
	*r*	*P*	*r*	*P*	*r*	*P*	*r*	*P*	*r*	*P*	*r*	*P*
Age	0.088	0.286	0.166	0.043	0.280	<0.001	0.274	<0.001	0.124	0.131	0.072	0.384
BMI	0.232	0.004	0.114	0.165	-0.067	0.416	-0.053	0.520	0.170	0.038	0.338	<0.001
Systolic BP	0.219	0.007	0.104	0.206	-0.032	0.703	0.058	0.479	-0.012	0.888	0.056	0.495
Diastolic BP	0.037	0.652	-0.084	0.311	-0.152	0.066	-0.103	0.211	-0.066	0.425	-0.018	0.829
GFR	-0.159	0.053	-0.106	0.197	-0.165	0.044	-0.110	0.180	-0.004	0.965	-0.086	0.298
Cortisol 8 AM	0.032	0.710	0.093	0.278	0.040	0.639	0.100	0.243	-0.042	0.624	-0.022	0.796
Cortisol 3 PM	0.030	0.761	0.025	0.799	0.209	0.031	0.182	0.059	-0.078	0.424	0.126	0.195
Cortisol 12 AM	0.070	0.474	0.158	0.105	0.175	0.073	0.311	0.001	0.067	0.494	-0.103	0.291
AUC-cortisol	0.045	0.589	0.194	0.018	0.265	0.001	0.178	0.030	0.040	0.625	0.051	0.535
DST-cortisol	0.031	0.705	0.281	<0.001	0.317	<0.001	0.376	<0.001	0.018	0.829	-0.014	0.870
Urinary cortisol	-0.152	0.064	-0.134	0.104	0.120	0.146	-0.065	0.434	0.152	0.065	0.044	0.595

BMI, body mass index; BP, blood pressure; GFR, glomerular filtration rate as assessed by 24-hour creatinine clearance; AUC-cortisol, area under the curve of daily plasma cortisol (8 AM, 3 PM, 12 AM); DST-cortisol, plasma cortisol after dexamethasone suppression test.

Multivariate regression analysis was conducted including different hemostatic markers as the dependent variables and age, sex, systolic blood pressure, creatinine clearance, and either AUC-cortisol (Model 1) or DST-cortisol (Model 2) as the independent variables, respectively (Supplementary Material). In both models, age was independently and directly related with F1 + 2 and vWF, and sex was independently related with log D-dimer. Both AUC-cortisol and DST-cortisol were significantly and independently correlated with log D-dimer (β-coefficient 0.001, P=0.029; β-coefficient 0.003, P<0.001; respectively), F1 + 2 (β-coefficient 0.267, P=0.002; β-coefficient 1.217, P<0.001; respectively), and vWF (β-coefficient 0.106, P=0.035; β-coefficient 0.877, P<0.001; respectively).

## Discussion

A prothrombotic state is associated with major cardiovascular events in hypertension, and many factors can contribute to hemostatic activation in hypertensive patients (37). Evidence previously obtained in patients with Cushing’s syndrome indicates that excess cortisol could contribute to a hypercoagulable state. We tested the hypothesis that even minor differences in regulation of cortisol secretion are associated with a prothrombotic state in patients with essential hypertension. Results show that plasma levels of D-dimer, F1 + 2, and vWF are progressively greater with increasing levels of plasma cortisol daily profile and response to DST. Both AUC-cortisol and DST-cortisol are significantly correlated with plasma D-dimer, F1 + 2, and vWF levels independently of age, BMI, blood pressure, and renal function. These findings indicate that differences in regulation of cortisol production within the physiologic range might contribute to a prothrombotic state in patients with hypertension.

Hypercortisolism is associated with high risk of cardiovascular morbidity and mortality that might be related to a multiplicity of factors (38) including a prothrombotic state. After initial studies that suggested presence of a hypercoagulable state in patients with Cushing’s syndrome (39), spontaneous activation of the hemostatic system was consistently shown in patients with hypercortisolism. Increased circulating levels of fibrinogen (19, 20, 40, 41), D-dimer (20, 23, 40), vWF (20, 42–44), AT-III (19–21, 44, 45), protein C-protein S complex (21, 22, 41, 45), and PAI-1 (19, 20, 41) were reported together with changes of additional coagulation factors and hemostatic tests (19–21, 41, 43, 46) in several cross-sectional comparisons of patients with Cushing’s syndrome with healthy subjects. Some studies suggested that these changes could be more relevant in patients with ACTH-producing adenomas (44) and increased levels of procoagulants and antifibrinolytics were reported also in children with Cushing’s syndrome that resolved after surgical treatment (45). Reversal of hypercoagulability was reported also in adults after surgical resolution of disease (43, 46), although this was not confirmed in other studies (40, 41). Interestingly, hemostatic changes were reported also in patients with excess plasma cortisol or incidentally detected adrenal masses in the absence of overt clinical features of hypercortisolism (22, 23), further supporting the hypothesis of a contribution of cortisol levels to regulation of coagulation and fibrinolysis (18). In our highly selected hypertensive patients who had physiologic suppression of plasma cortisol after DST, cortisol levels were significantly and independently associated with markers of intrinsic hemostatic activation. This study expands to a physiologic range of hormonal secretion the evidence of the potential role of cortisol in causing a prothrombotic state.

Due to its high prevalence in the general population, arterial hypertension is considered the leading cardiovascular risk factor. In patients with essential hypertension, we have previously demonstrated that a prothrombotic state contributes to the development of target organ damage and thereby might increase cardiovascular morbidity and mortality (7, 9–15). In fact, substantial experimental and clinical data indicate that platelets and coagulation cascade are important determinants of both atherogenesis and atherothrombosis (1). The hemostatic system exerts several actions on the vasculature that could influence the structure of the arterial wall and presumably the progression of atherosclerotic lesions. Hemostatic components have been implicated in causing disruption of endothelial lining, leukocyte recruitment, oxidative stress, vascular inflammation, migration of vascular smooth muscle cells, apoptosis, and angiogenesis (47, 48). For these reasons, identification of conditions that may contribute to a prothrombotic state in hypertension would be relevant as an issue for possible preventive interventions.

Many factors could cause activation of the hemostatic system in hypertension and the present study demonstrates that even minor differences in regulation of cortisol secretion are associated with a prothrombotic state as defined by higher levels of D-dimer, F1 + 2, and vWF. Measurements of fibrin D-dimer, the principal breakdown fragment of fibrin, and F1 + 2 that is released when coagulation factor Xa converts prothrombin to thrombin provide a reliable estimation of the overall state of activation of the coagulation pathways. On the other hand, vWF enhances adhesion of platelets to subendothelial collagen and increases the stability of coagulation factor VIII in the hemostatic cascade. Older age, obesity, and impaired renal function are associated with a prothrombotic state, and this is why statistical analysis was corrected for these variables showing that D-dimer, F1 + 2, and vWF were all independently related to both AUC-cortisol and DST-cortisol. Thus, differences in cortisol secretion within a physiological range could contribute to a prothrombotic state and thereby to hypertensive organ damage. Also, to this point it should be noticed that minor differences in regulation of cortisol secretion were previously found to be associated with impaired glucose metabolism in nondiabetic hypertensive patients (36) and greater left ventricular mass (30), suggesting additional mechanisms that might mediate detrimental cardiovascular effects of cortisol in essential hypertension.

Mechanisms that could link excess cortisol secretion to the hypercoagulable state are mostly speculative. Cortisol-induced up-regulation of gene transcription of multiple coagulation factors with intrinsic activation of the hemostatic cascade is the most likely mechanism. Activation of markers of the overall activity of the coagulation system such as D-dimer and F1 + 2 would support this possibility. On the other hand, observation of significantly increased levels of vWF might suggest that the prothrombotic state caused by increased circulating cortisol could be related to an enhanced metabolic function of endothelial cells. This was also suggested by Fatti et al. who reported increased levels of additional markers of endothelial activation (thrombin-antithrombin complex and plasmin-antiplasmin complex) in patients with Cushing’s syndrome (43). Hypercortisolism may also affect the multimeric structure of vWF causing an overexpression of abnormally high molecular weight multimers, capable of inducing spontaneous platelet aggregation (42).

Limitations to the present study need to be considered. First, the cross-sectional design limits the possibility to establish causality of the relationship between cortisol secretion and the prothrombotic state, although independence of this relationship from confounders in the multivariate analysis would suggest so. However, statistical adjustments cannot account for all biological and pathophysiological variables. Also, the possibility that plasma cortisol levels and prothrombotic markers causally affect one another cannot be excluded. Second, use of a selected clinic sample of white Caucasian hypertensive patients might limit the possibility to extend the present findings to a broader context. Third, inclusion of a significant proportion of patients who had been treated with antihypertensive drugs might have affected the results. However, it must be noticed that no differences were found in either hemostatic markers or cortisol measurements between these patients and those who were treatment naïve, nor differences were observed among patients who were treated with different categories of antihypertensive drugs. Last, due to the relatively small size of this study, the possibility that associations are merely due to chance should be considered.

## Conclusion

This study demonstrates for the first time that even minor differences in daily cortisol secretion and cortisol levels after overnight suppression are independently associated with markers of a prothrombotic state in patients with essential hypertension who are free of major cardiovascular and renal complications. These findings expand this evidence beyond the boundary of clinical and subclinical Cushing’s syndrome. Results also provide better knowledge of factors that might contribute to develop a prothrombotic state in hypertension, thereby increasing target organ damage and, in turn, the risk of cardiovascular events. This study might have important clinical implications opening new paths to the possibility to identify those hypertensive patients at higher risk of development of subclinical organ damage. Detection of cortisol levels in the upper limit of normality in conjunction with markers of hemostatic activation could be useful to guide physicians toward more aggressive treatment and control of blood pressure and additional risk factors. Also, these findings open a window to the possibility to effectively prevent hypertensive organ damage through specific interventions on cortisol production and hemostatic system. Possible benefits of these interventions will have to be tested in future studies.

## Data availability statement

The raw data supporting the conclusions of this article will be made available by the authors, without undue reservation.

## Ethics statement

The studies involving humans were approved by Institutional Review Board - Department of Medicine - University of Udine. The studies were conducted in accordance with the local legislation and institutional requirements. The participants provided their written informed consent to participate in this study.

## Author contributions

GB: Conceptualization, Data curation, Formal analysis, Investigation, Methodology, Supervision, Writing – original draft. AD: Conceptualization, Data curation, Formal analysis, Investigation, Methodology, Supervision, Writing – original draft. LB: Data curation, Investigation, Methodology, Software, Supervision, Validation, Visualization, Writing – review & editing. AV: Data curation, Investigation, Methodology, Software, Supervision, Validation, Visualization, Writing – review & editing. NB: Data curation, Investigation, Supervision, Validation, Visualization, Writing – review & editing. CV: Data curation, Investigation, Supervision, Validation, Visualization, Writing – review & editing. CC: Conceptualization, Data curation, Formal analysis, Funding acquisition, Investigation, Methodology, Project administration, Resources, Supervision, Validation, Writing – original draft, Writing – review & editing. LS: Conceptualization, Data curation, Formal analysis, Funding acquisition, Investigation, Methodology, Project administration, Resources, Supervision, Validation, Writing – original draft, Writing – review & editing.
