# Supplementary material for: Daytime plasma cortisol and cortisol response to dexamethasone suppression are associated with a prothrombotic state in hypertension
Source: Front Endocrinol (Lausanne). 2024 May 21;15:1397062. doi: 10.3389/fendo.2024.1397062 (PMC11148267; doi:10.3389/fendo.2024.1397062)
Supplement: Supplementary file 1 [file Table_1.docx]

Supplementary Material

Daytime Plasma Cortisol and Cortisol Response to Dexamethasone Suppression Are Associated with a Prothrombotic State in Hypertension

**Gabriele Brosolo^1^, Andrea Da Porto^2^, Luca Bulfone^1^, Antonio Vacca^1^, Nicole Bertin^3^, Cinzia Vivarelli^1^, Alessandro Frangipane^1^, Cristiana Catena^1†^, Leonardo A. Sechi^1,2,3*†^,**

^1^Internal Medicine and European Hypertension Excellence Center, Department of Medicine, University of Udine, Udine, Italy

^2^Diabetes and Metabolism Unit, Department of Medicine, University of Udine, Udine, Italy

^3^Thrombosis and Hemostasis Unit, Department of Medicine, University of Udine, Udine, Italy

**^†^**These authors contributed equally to this work and share last authorship

*** Correspondence**

Corresponding author:

Leonardo A. Sechi, MD, FACP, EHS

Clinica Medica, Department of Medicine

University Hospital, Building 8

33100-Udine, Italy

Phone +39 0432 559804

Fax +39 0432 559490

Email sechi@uniud.it

# Supplementary Tables

**Multivariate Correlation with log D-dimer as the Dependent Variable**

**(Model 1 includes AUC-cortisol, Model 2 includes DST-cortisol as independent variables)**

| **Model 1** | |  |  |  |  |  |  |  |
| --- | --- | --- | --- | --- | --- | --- | --- | --- |
|  |  |  |  |  |  |  |  |  |
| *Statistics of regression* | |  |  |  |  |  |  |  |
| Multiple R | 0,33784847 |  |  |  |  |  |  |  |
| Squared R | 0,11414159 |  |  |  |  |  |  |  |
| Squared R corrected | 0,07671095 |  |  |  |  |  |  |  |
| Standard error | 0,19167004 |  |  |  |  |  |  |  |
| Observations | 149 |  |  |  |  |  |  |  |
|  |  |  |  |  |  |  |  |  |
| ANALYSIS OF VARIANCE | |  |  |  |  |  |  |  |
|  | *df* | *SQ* | *MQ* | *F* | *Significance F* |  |  |  |
| Regression | 6 | 0,6721658 | 0,11202763 | 3,04941611 | 0,00776743 |  |  |  |
| Residual | 142 | 5,21671144 | 0,0367374 |  |  |  |  |  |
| Total | 148 | 5,88887723 |  |  |  |  |  |  |
|  |  |  |  |  |  |  |  |  |
|  | *Coefficients* | *Standard error* | *Stat t* | *Significance* | *Inferior 95%* | *Superior 95%* | *Inferior 95,0%* | *Superior 95,0%* |
| intercept | 1,95205669 | 0,19788508 | 9,86459771 | 8,3628E-18 | 1,56087529 | 2,34323809 | 1,56087529 | 2,34323809 |
| age | 0,00173353 | 0,0012503 | 1,38649045 | 0,16777018 | -0,0007381 | 0,00420514 | -0,0007381 | 0,00420514 |
| sex | -0,0802973 | 0,03180008 | -2,525067 | 0,01266635 | -0,1431601 | -0,0174346 | -0,1431601 | -0,0174346 |
| body mass index | 0,00222995 | 0,00282581 | 0,7891358 | 0,43134825 | -0,0033561 | 0,00781605 | -0,0033561 | 0,00781605 |
| systolic blood pressure | 0,00126753 | 0,00093163 | 1,36054969 | 0,17581173 | -0,0005741 | 0,0031092 | -0,0005741 | 0,0031092 |
| creatinine clearance | -6,356E-05 | 0,00064786 | -0,0981144 | 0,9219798 | -0,0013443 | 0,00121713 | -0,0013443 | 0,00121713 |
| AUC-cortisol | 0,00048228 | 0,00021807 | 2,21157626 | 0,02859443 | 5,1195E-05 | 0,00091336 | 5,1195E-05 | 0,00091336 |

| **Model 2** | |  |  |  |  |  |  |  |
| --- | --- | --- | --- | --- | --- | --- | --- | --- |
|  |  |  |  |  |  |  |  |  |
| *Statistics of regression* | |  |  |  |  |  |  |  |
| Multiple R | 0,39524003 |  |  |  |  |  |  |  |
| Squared R | 0,15621468 |  |  |  |  |  |  |  |
| Squared R corrected | 0,12056178 |  |  |  |  |  |  |  |
| Standard error | 0,18706307 |  |  |  |  |  |  |  |
| Observations | 149 |  |  |  |  |  |  |  |
|  |  |  |  |  |  |  |  |  |
| ANALYSIS OF VARIANCE | |  |  |  |  |  |  |  |
|  | *df* | *SQ* | *MQ* | *F* | *Significance F* |  |  |  |
| Regression | 6 | 0,9199291 | 0,15332152 | 4,38154208 | 0,00043095 |  |  |  |
| Residual | 142 | 4,96894813 | 0,03499259 |  |  |  |  |  |
| Total | 148 | 5,88887723 |  |  |  |  |  |  |
|  |  |  |  |  |  |  |  |  |
|  | *Coefficients* | *Standard error* | *Stat t* | *Significance* | *Inferior 95%* | *Superior 95%* | *Inferior 95,0%* | *Superior 95,0%* |
| intercept | 1,98743363 | 0,18659641 | 10,6509745 | 7,8011E-20 | 1,61856779 | 2,35629947 | 1,61856779 | 2,35629947 |
| age | 0,00137718 | 0,00122566 | 1,12362526 | 0,26306803 | -0,0010457 | 0,00380008 | -0,0010457 | 0,00380008 |
| sex | -0,0740697 | 0,03088266 | -2,3984239 | 0,01776455 | -0,1351189 | -0,0130205 | -0,1351189 | -0,0130205 |
| body mass index | 0,00422322 | 0,00271804 | 1,55377422 | 0,12246423 | -0,0011498 | 0,00959628 | -0,0011498 | 0,00959628 |
| systolic blood pressure | 0,00110077 | 0,00090899 | 1,21098346 | 0,22791334 | -0,0006961 | 0,00289766 | -0,0006961 | 0,00289766 |
| creatinine clearance | -0,0003187 | 0,00060961 | -0,5228342 | 0,60190446 | -0,0015238 | 0,00088637 | -0,0015238 | 0,00088637 |
| DST-cortisol | 0,00305138 | 0,00087306 | 3,49505359 | 0,00063264 | 0,00132551 | 0,00477725 | 0,00132551 | 0,00477725 |

**Multivariate Correlation with Prothrombin Fragment 1+2 as the Dependent Variable**

**(Model 1 includes AUC-cortisol, Model 2 includes DST-cortisol as independent variables)**

| **Model 1** | |  |  |  |  |  |  |  |
| --- | --- | --- | --- | --- | --- | --- | --- | --- |
|  |  |  |  |  |  |  |  |  |
| *Statistics of regression* | |  |  |  |  |  |  |  |
| Multiple R | 0,41089449 |  |  |  |  |  |  |  |
| Squared R | 0,16883428 |  |  |  |  |  |  |  |
| Squared R corrected | 0,1337146 |  |  |  |  |  |  |  |
| Standard error | 73,0661919 |  |  |  |  |  |  |  |
| Observations | 149 |  |  |  |  |  |  |  |
|  |  |  |  |  |  |  |  |  |
| ANALYSIS OF VARIANCE | |  |  |  |  |  |  |  |
|  | *df* | *SQ* | *MQ* | *F* | *Significance F* |  |  |  |
| Regression | 6 | 153990,631 | 25665,1052 | 4,80739827 | 0,00017043 |  |  |  |
| Residual | 142 | 758090,912 | 5338,6684 |  |  |  |  |  |
| Total | 148 | 912081,544 |  |  |  |  |  |  |
|  |  |  |  |  |  |  |  |  |
|  | *Coefficients* | *Standard error* | *Stat t* | *Significance* | *Inferior 95%* | *Superior 95%* | *Inferior 95,0%* | *Superior 95,0%* |
| intercept | 204,490717 | 75,4354153 | 2,71080522 | 0,00754015 | 55,3691592 | 353,612276 | 55,3691592 | 353,612276 |
| age | 1,66762354 | 0,47662517 | 3,49881552 | 0,00062448 | 0,72542567 | 2,60982142 | 0,72542567 | 2,60982142 |
| sex | 0,06882586 | 12,1224496 | 0,00567755 | 0,99547796 | -23,894966 | 24,0326175 | -23,894966 | 24,0326175 |
| body mass index | -2,2634522 | 1,0772231 | -2,1011917 | 0,03739087 | -4,3929187 | -0,1339858 | -4,3929187 | -0,1339858 |
| systolic blood pressure | -0,3767321 | 0,35514641 | -1,0607797 | 0,29059093 | -1,0787894 | 0,32532524 | -1,0787894 | 0,32532524 |
| creatinine clearance | -0,17914 | 0,2469698 | -0,7253519 | 0,46942983 | -0,6673526 | 0,30907259 | -0,6673526 | 0,30907259 |
| AUC-cortisol | 0,26652875 | 0,08312963 | 3,20618244 | 0,0016619 | 0,10219719 | 0,43086031 | 0,10219719 | 0,43086031 |

| **Model 2** | |  |  |  |  |  |  |  |
| --- | --- | --- | --- | --- | --- | --- | --- | --- |
|  |  |  |  |  |  |  |  |  |
| *Statistics of regression* | |  |  |  |  |  |  |  |
| Multiple R | 0,42803239 |  |  |  |  |  |  |  |
| Squared R | 0,18321173 |  |  |  |  |  |  |  |
| Squared R corrected | 0,14869955 |  |  |  |  |  |  |  |
| Standard error | 72,4314881 |  |  |  |  |  |  |  |
| Observations | 149 |  |  |  |  |  |  |  |
|  |  |  |  |  |  |  |  |  |
| ANALYSIS OF VARIANCE | |  |  |  |  |  |  |  |
|  | *df* | *SQ* | *MQ* | *F* | *Significance F* |  |  |  |
| Regression | 6 | 167104,037 | 27850,6728 | 5,30861067 | 5,7401E-05 |  |  |  |
| Residual | 142 | 744977,507 | 5246,32047 |  |  |  |  |  |
| Total | 148 | 912081,544 |  |  |  |  |  |  |
|  |  |  |  |  |  |  |  |  |
|  | *Coefficients* | *Standard error* | *Stat t* | *Significance* | *Inferior 95%* | *Superior 95%* | *Inferior 95,0%* | *Superior 95,0%* |
| intercept | 238,253643 | 72,2507966 | 3,29759192 | 0,00123231 | 95,4274741 | 381,079812 | 95,4274741 | 381,079812 |
| age | 1,53386405 | 0,47457991 | 3,23204587 | 0,00152801 | 0,59570926 | 2,47201884 | 0,59570926 | 2,47201884 |
| sex | 3,62836777 | 11,957877 | 0,30342909 | 0,76200699 | -20,010095 | 27,2668304 | -20,010095 | 27,2668304 |
| body mass index | -1,2866567 | 1,0524353 | -1,2225519 | 0,2235243 | -3,3671224 | 0,79380894 | -3,3671224 | 0,79380894 |
| systolic blood pressure | -0,4549863 | 0,35196226 | -1,2927133 | 0,19820965 | -1,1507492 | 0,24077656 | -1,1507492 | 0,24077656 |
| creatinine clearance | -0,3409275 | 0,23604486 | -1,4443337 | 0,15084801 | -0,8075436 | 0,12568854 | -0,8075436 | 0,12568854 |
| DST-cortisol | 1,21698779 | 0,33805092 | 3,60001321 | 0,00043885 | 0,54872501 | 1,88525058 | 0,54872501 | 1,88525058 |

**Multivariate Correlation vonWillebrand factor as the Dependent Variable**

**(Model 1 includes AUC-cortisol, Model 2 includes DST-cortisol as independent variables)**

| **Model 1** | |  |  |  |  |  |  |  |
| --- | --- | --- | --- | --- | --- | --- | --- | --- |
|  |  |  |  |  |  |  |  |  |
| *Statistics of regression* | |  |  |  |  |  |  |  |
| Multiple R | 0,34009312 |  |  |  |  |  |  |  |
| Squared R | 0,11566333 |  |  |  |  |  |  |  |
| Squared R corrected | 0,07829699 |  |  |  |  |  |  |  |
| Standard error | 43,7140916 |  |  |  |  |  |  |  |
| Observations | 149 |  |  |  |  |  |  |  |
|  |  |  |  |  |  |  |  |  |
| ANALYSIS OF VARIANCE | |  |  |  |  |  |  |  |
|  | *df* | *SQ* | *MQ* | *F* | *Significance F* |  |  |  |
| Regression | 6 | 35490,2717 | 5915,04529 | 3,09538846 | 0,00703728 |  |  |  |
| Residual | 142 | 271350,896 | 1910,9218 |  |  |  |  |  |
| Total | 148 | 306841,168 |  |  |  |  |  |  |
|  |  |  |  |  |  |  |  |  |
|  | *Coefficients* | *Standard error* | *Stat t* | *Significance* | *Inferior 95%* | *Superior 95%* | *Inferior 95,0%* | *Superior 95,0%* |
| intercept | 82,9492711 | 45,1315522 | 1,83794412 | 0,06816048 | -6,2672774 | 172,16582 | -6,2672774 | 172,16582 |
| age | 0,92420131 | 0,28515563 | 3,24104172 | 0,00148384 | 0,36050251 | 1,4879001 | 0,36050251 | 1,4879001 |
| sex | -2,1893357 | 7,25262748 | -0,3018679 | 0,76319439 | -16,526409 | 12,147738 | -16,526409 | 12,147738 |
| body mass index | -0,95589 | 0,64448178 | -1,4831916 | 0,1402396 | -2,2299086 | 0,31812869 | -2,2299086 | 0,31812869 |
| systolic blood pressure | 0,03008787 | 0,21247724 | 0,14160516 | 0,88759259 | -0,3899395 | 0,45011522 | -0,3899395 | 0,45011522 |
| creatinine clearance | -0,0534919 | 0,14775726 | -0,3620254 | 0,71787122 | -0,3455801 | 0,23859629 | -0,3455801 | 0,23859629 |
| AUC-cortisol | 0,10584701 | 0,04973485 | 2,12822621 | 0,03504501 | 0,00753061 | 0,20416341 | 0,00753061 | 0,20416341 |

| **Model 2** | |  |  |  |  |  |  |  |
| --- | --- | --- | --- | --- | --- | --- | --- | --- |
|  |  |  |  |  |  |  |  |  |
| *Statistics of regression* | |  |  |  |  |  |  |  |
| Multiple R | 0,45004711 |  |  |  |  |  |  |  |
| Squared R | 0,2025424 |  |  |  |  |  |  |  |
| Squared R corrected | 0,16884701 |  |  |  |  |  |  |  |
| Standard error | 41,5113103 |  |  |  |  |  |  |  |
| Observations | 149 |  |  |  |  |  |  |  |
|  |  |  |  |  |  |  |  |  |
| ANALYSIS OF VARIANCE | |  |  |  |  |  |  |  |
|  | *df* | *SQ* | *MQ* | *F* | *Significance F* |  |  |  |
| Regression | 6 | 62148,346 | 10358,0577 | 6,010982171 | 1,2626E-05 |  |  |  |
| Residual | 142 | 244692,822 | 1723,18889 |  |  |  |  |  |
| Total | 148 | 306841,168 |  |  |  |  |  |  |
|  |  |  |  |  |  |  |  |  |
|  | *Coefficients* | *Standard error* | *Stat t* | *Significance* | *Inferior 95%* | *Superior 95%* | *Inferior 95,0%* | *Superior 95,0%* |
| intercept | 84,4350571 | 41,407754 | 2,03911222 | 0,043293746 | 2,57975382 | 166,29036 | 2,57975382 | 166,29036 |
| age | 0,81808235 | 0,27198715 | 3,00779778 | 0,0031136 | 0,28041516 | 1,35574954 | 0,28041516 | 1,35574954 |
| sex | -0,8746093 | 6,8531954 | -0,1276207 | 0,898629734 | -14,422081 | 12,6728627 | -14,422081 | 12,6728627 |
| body mass index | -0,4632923 | 0,60316265 | -0,7681051 | 0,443700231 | -1,6556308 | 0,72904627 | -1,6556308 | 0,72904627 |
| systolic blood pressure | -0,0126584 | 0,20171358 | -0,0627545 | 0,950050224 | -0,4114081 | 0,38609118 | -0,4114081 | 0,38609118 |
| creatinine clearance | -0,1003167 | 0,13528 | -0,7415487 | 0,4595855 | -0,3677397 | 0,16710628 | -0,3677397 | 0,16710628 |
| DST-cortisol | 0,87704905 | 0,19374083 | 4,52691897 | 1,25602E-05 | 0,49406004 | 1,26003806 | 0,49406004 | 1,26003806 |
